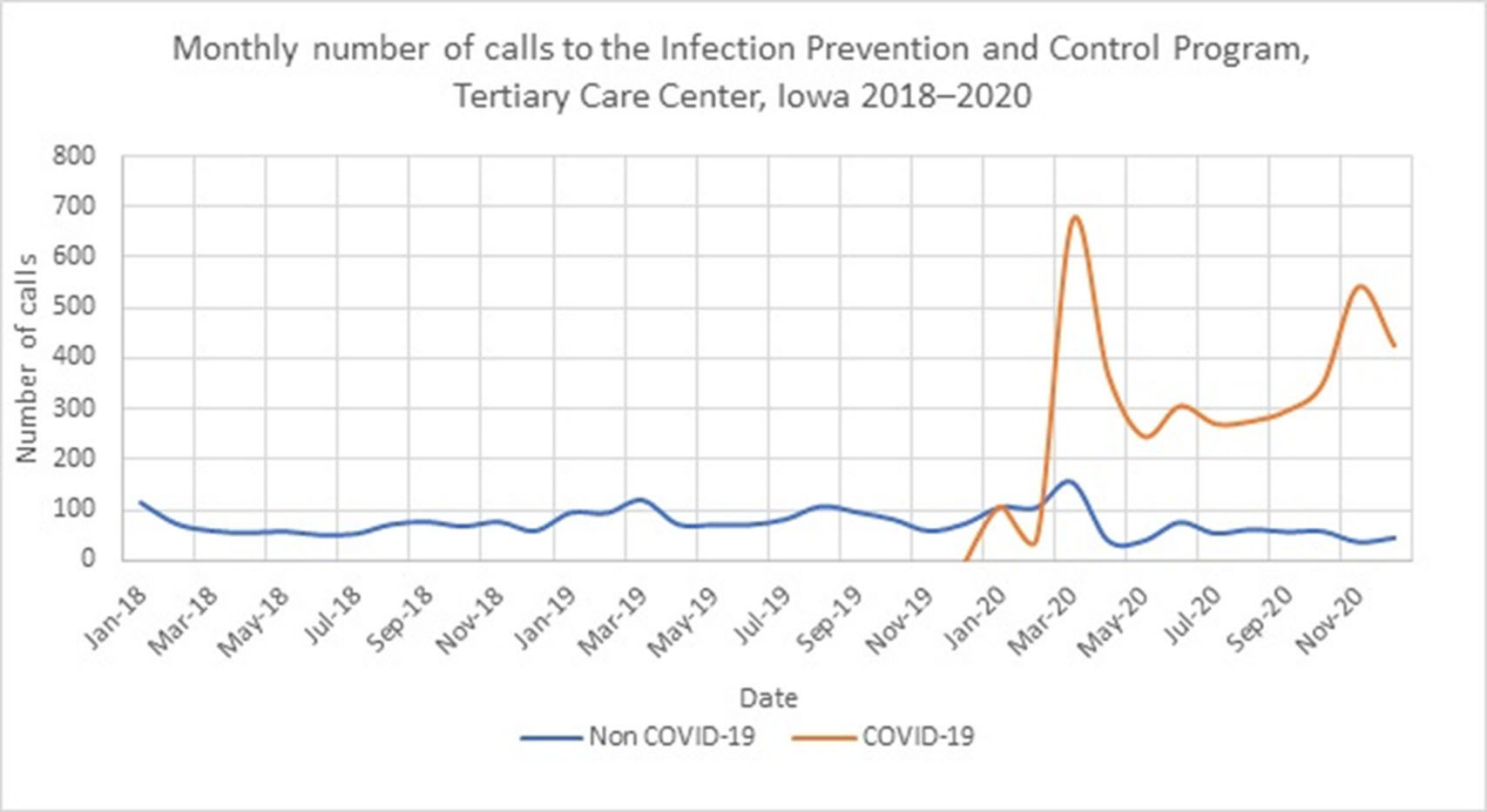# Impact of COVID-19 on Volume of Infection Prevention and Control Calls at a Tertiary-Care Center in Iowa, 2018–2020

**DOI:** 10.1017/ash.2021.103

**Published:** 2021-07-29

**Authors:** Mohammed Alsuhaibani, Takaaki Kobayashi, Stephanie Holley, Angie Dains, Oluchi Abosi, Kyle Jenn, Holly Meacham, Lorinda Sheeler, William Etienne, Alexandra Trannel, Mary Kukla, Alexandre Marra, Melanie Wellington, Daniel Diekema, Jorge Salinas

## Abstract

**Background:** The COVID-19 pandemic has affected healthcare systems worldwide, but the impact on infection prevention and control (IPC) programs has not been fully evaluated. We assessed the impact of the COVID-19 pandemic on IPC consultation requests. **Methods:** The University of Iowa Hospitals & Clinics comprises an 811-bed hospital that admits >36,000 patients yearly and >200 outpatient clinics. Questions about IPC can be addressed to the Program of Hospital Epidemiology via e-mail, in person, or through our phone line. We routinely record date and time, call source, reason for the call, and estimated time to resolve questions for all phone line requests. We defined calls during 2018–2019 as the pre–COVID-19 period and calls from January to December 2020 as the COVID-19 period. **Results:** In total, 6,564 calls were recorded from 2018 to 2020. In the pre–COVID-19 period (2018–2019), we received a median of 71 calls per month (range, 50–119). The most frequent call sources were inpatient units (n = 902; 50%), department of public health (n = 357; 20%), laboratory (n = 171; 9%), and outpatient clinics (n = 120; 7%) (Figure 1). The most common call topics were isolation and precautions (n = 606; 42%), outside institutions requests (n = 324; 22%), environmental and construction (n = 148; 10%), and infection exposures (n = 149; 10%). The most frequent infection-related calls were about tuberculosis (17%), gram-negative organisms (14%), and influenza (9%). During the COVID-19 period, the median monthly call volume increased 500% to 368 per month (range, 149–829). Most (83%) were COVID-19 related. The median monthly number of COVID-19 calls was 302 (range, 45–674). The median monthly number of non–COVID-19 calls decreased to 56 (range, 36–155). The most frequent call sources were inpatient units (57%), outpatient clinics (16%), and the department of public health (5%). Most calls concerned isolation and precautions (50%) and COVID-19 testing (20%). The mean time required to respond to each question was 10 minutes (range, 2–720). The biggest surges in calls during the COVID-19 period were at the beginning of the pandemic (March 2020) and during the hospital peak COVID-19 census (November 2020). **Conclusions:** In addition to supporting a proactive COVID-19 response, our IPC program experienced a 500% increase in consultation requests. Planning for future bioemergencies should include creative strategies to provide additional resources to increase response capacity within IPC programs.

**Funding:** No

**Disclosures:** None

**Figure 1.**